# A multi-method assessment of 3D printed micromorphological osteological features

**DOI:** 10.1007/s00414-022-02789-y

**Published:** 2022-02-09

**Authors:** Rachael M. Carew, Francesco Iacoviello, Carolyn Rando, Robert M. Moss, Robert Speller, James French, Ruth M. Morgan

**Affiliations:** 1 UCL Department of Security and Crime Science, University College London, 35 Tavistock Square, London, WC1H 9EZ UK; 2 UCL Centre for the Forensic Sciences, University College London, 35 Tavistock Square, London, WC1H 9EZ UK; 3 The Electrochemical Innovation Lab, Department of Chemical Engineering, University College London, London, UK; 4 UCL Institute of Archaeology, University College London, 31-34 Gordon Square, London, WC1H 0PY UK; 5 UCL Department of Medical Physics & Biomedical Engineering, University College London, Gower Street, London, WC1E 6BT UK

**Keywords:** Forensic anthropology, 3D imaging, 3D modelling, 3D printing, Evidence reconstruction, Trauma

## Abstract

The evaluation of 3D printed osteological materials has highlighted the difficulties associated with accurately representing fine surface details on printed bones. Moreover, there is an increasing need for reconstructions to be demonstrably accurate and reliable for use in the criminal justice system. The aim of this study was to assess the surface quality of 3D prints (*n* = 9) that presented with micromorphological alterations from trauma, taphonomy and pathology processes. The archaeological bones were imaged using micro-CT scanning and 3D printed with selective laser sintering (SLS) printing. A multi-method experimental approach subsequently identified: (1) the 3D printed bones to be metrically accurate to within 1.0 mm; (2) good representation of micromorphological surface features overall, albeit with some loss of intricate details, depths, and fine textures that can be important for visual processing; (3) five of the nine 3D printed bones were quantitatively scored as accurate using the visual comparison method; and, (4) low mesh comparison distances (± 0.2 mm) between the original models and the digitised 3D print models. The findings offer empirical data that can be used to underpin 3D printed reconstructions of exhibits for use in courts of law. In addition, an adaptable pathway was presented that can be used to assess 3D print accuracy in future reconstructions.

## Introduction

The use of 3D models and prints in forensic science can be beneficial for demonstration purposes in a courtroom setting [1, 2]. 3D printed osteological replicas have been examined for their accuracy [3, 4] and are increasingly being used in a wide range of forensic applications [5–10]. Osteological samples can present with fine micromorphological surface details that can be important for evaluative interpretation of forensic events and external processes such as from trauma, taphonomy, and pathology. The presentation of micromorphological details can therefore enhance understanding of, for example, mechanisms of injury; however, previous research using CT data has demonstrated difficulty in obtaining accurately 3D printed micromorphological details [11]. This study therefore investigated the accuracy of 3D printed bones that exhibited a range of micromorphological surface details (approximately less than 1 mm), utilising micro-computed tomography (micro-CT) scanning using a multi-method experimental approach that included a computational mesh comparison.

## Background

A preliminary investigation into the accuracy of 3D printing bone replicas from CT scans by Carew et al. [4], tested six different 3D printers and identified a range (± 2.0 mm) that printed reconstructions should fall within to be considered accurate, this range originated from the generally accepted range for error in forensic anthropology [12]. Additionally, a number of recommendations for obtaining accurate prints were offered, including the use of Selective Laser Sintering (SLS) [4]. Subsequent research has also assessed the surface quality of printed bones using SLS printing and found that not all surface details were well represented [11]. The complex nature of bone structure and highly textural features of bone surfaces can be challenging to retain at sufficient levels in 3D printed replicas. Carew et al. [11] suggested that micro-CT may be a more suitable digital capture method for scanning bones where high surface detail is required, due to the greater spatial resolution obtained from micro-CT scanning and its ability to capture fine details, such as cut marks [3].

Micro-CT is a non-invasive, transmissive scan method that captures volumetric data using x-radiation of a sample that rotates within the scan chamber [13]. Its value in casework has been presented for histological analysis such as species identification [14], for soft and hard tissue examination in forensic pathology [15], for physical fit analysis of bone fragments [16], and for cutmark analysis [3, 17]. The analysis of micromorphological details is important for evaluative interpretation in forensic casework in terms of indicating possible trauma (e.g., cutmarks or striations), taphonomy (e.g., heat induced bones fractures), or pathology (e.g., evidence of disease or malnutrition). Micro-CT has the potential to capture these details due to its high spatial resolution and greater image quality compared to clinical CT [18].

The detailed capture and representation of fine details from trauma pose a new challenge for crime reconstructions. Cranial fracture lines have been replicated through 3D printing with limited success; studies have found that fractures from blunt force trauma captured using surface scanning techniques [19] and from gunshot trauma from post-mortem computed tomography [20] could only be partially observed. Experimental research using micro-CT to capture cutmarks on porcine bone found the 3D printed replica bones to be accurate to the original bones to < 0.62 mm for overall morphology and < 0.36 mm for toolmarks [3]. The models were 3D printed using three printers (Polyjet Material Jetting printer, Fused Deposition Modelling (FDM), and Stereolithography (SLA)), and rescanned using a surface scanner to perform a mesh comparison between the original models and printed models. Baier et al. [3] demonstrate the utility of the mesh comparison method for providing good quantitative results, but also offer caution against 3D printing important features that are smaller than 3 mm.

Little research has been identified that has examined 3D printed bones with taphonomy; however, 3D printed burnt bone fragments provided a useful tool for demonstrating the reconstruction and physical fit of fragments, which was also non-destructive and valuable for use with fragile fragments [16]. Both ante-mortem healed trauma and pathological lesions can also be important intelligence for identification purposes; however, few studies have investigated the printing of pathological specimens for crime reconstruction purposes. Intricate surface details from a calcified *leiomyoma* (a smooth muscle tumour) were reported to be well presented on a 3D printed replica [21]. The *leiomyoma* was captured using surface scanning and reconstructed using FDM printing, with the pathological changes appearing to be a good representation on the 3D print from the raised areas, but the recessed areas were not well captured due to the use of surface scanning [21]. Similarly, a scaled-up printed replica femur from a 19-week-old foetus that presented with *osteogenesis imperfecta* was reported to be a useful tool for demonstrating pathological features [22]. However, the use of scaling has not been investigated in 3D printing for crime reconstruction purposes.

While there is currently limited published research addressing the use of 3D printed bones exhibiting indicators of trauma, taphonomy, and pathology, the value of such prints has been highlighted [16, 19, 21, 22]. The accuracy of 3D printed bones is key to ensure that accurate and representative prints are produced, particularly if they were to be used within a court of law [3, 4, 11]. Printing errors can arise through the scanning, modelling, and/or printing steps and therefore each step used ought to be carefully considered and justified prior to implementation [20, 23]. Studies have investigated the effect of individual parameters in bone modelling and printing, such as during scanning [24], thresholding and segmentation [25], and generally for comparing medical models [23]. To ensure that printed replicas are of sufficient quality for the level of interpretation required by the expert or the jury, steps can be implemented to both mitigate against print errors and quantify print accuracy [3, 11]. Mesh comparison methods offer a comparison workflow that is independent of the observer measurement error found with manual measurements and provides a higher quantitative method for assessing print accuracy [3, 26]. Therefore, this study sought to assess the surface quality of 3D prints exhibiting alterations from trauma, taphonomy and pathology processes, to determine whether it was possible to observe accurate micromorphological details on 3D printed bones.

## Methods and materials

A multi-method experimental design was developed to evaluate the surface quality and accuracy of 3D printed bones that exhibited intricate surface details, utilising four approaches that allowed for a robust examination of the overall print accuracy. The multi-method assessment included the following four steps:

1. A quality control step, to check that no scaling errors had occurred;
2. A qualitative assessment, to visually inspect for similarities and differences between the original bones and the 3D prints;
3. An objective visual comparison, to quantitatively score the 3D print surfaces using the method from Edwards and Rogers [19];
4. A quantitative assessment, using a mesh comparison to assess the accuracy of the 3D prints, with the ideal range ± 1.0 mm [4, 11].

A single experienced observer performed each of the four assessment steps. The observer was a UK Level III Certified Forensic Anthropologist with 5 years’ experience of generating and assessing 3D printed bones. A single observer was used to reduce the possibility of observer bias and different experience levels from multiple observers affecting the results.

### Materials

Nine dry bones of archaeological origin were selected from the teaching collection in the Institute of Archaeology at UCL. These bones exhibited features from trauma, taphonomy and pathology processes, exhibited intricate micromorphological details, and had dimensions that permitted analysis inside the scan chamber (Table 1).

**Table 1:** Bones (A–I) with details of the bone, the surface features, and type of features present

Bone	Description	Surface features	Type
A	Non-human bone (distal)	Butchery, cut marks, woven bone; fracture lines	Trauma
B	Non-human (mid-shaft)	Chicken bone with rat toothmarks; exposed trabecular bone	Trauma
C	Non-human bone (mid-shaft)	Gnawed bone with toothmarks; flaking/depression fracture	Trauma
D	Non-human bone (mid-shaft)	Butchery, chop mark; exposed trabecular bone; very shallow root etching	Trauma
E	Human (*Homo sapiens*) right second metatarsal	Ante-mortem misaligned healed trauma with callus; macroporosity	Pathology
F	Non-human crania fragment	Taphonomy with root etchings; exposed diploë (spongy) bone; sutures and fractured edges	Taphonomy
G	Human (*Homo sapiens*) patella	Osteoarthritis: osteophytes, enthesophytes, woven bone, exostosis; eburnation, grooves (anterior); exposed trabecular bone	Pathology
H	Non-human fragment	Cremated calcined bone with transverse fractures (some light grey, mostly white)	Taphonomy
I	Non-human fragment	Cremated, partly calcined bone (dark grey and white)	Taphonomy

### Image acquisition

**Fig. 1:**
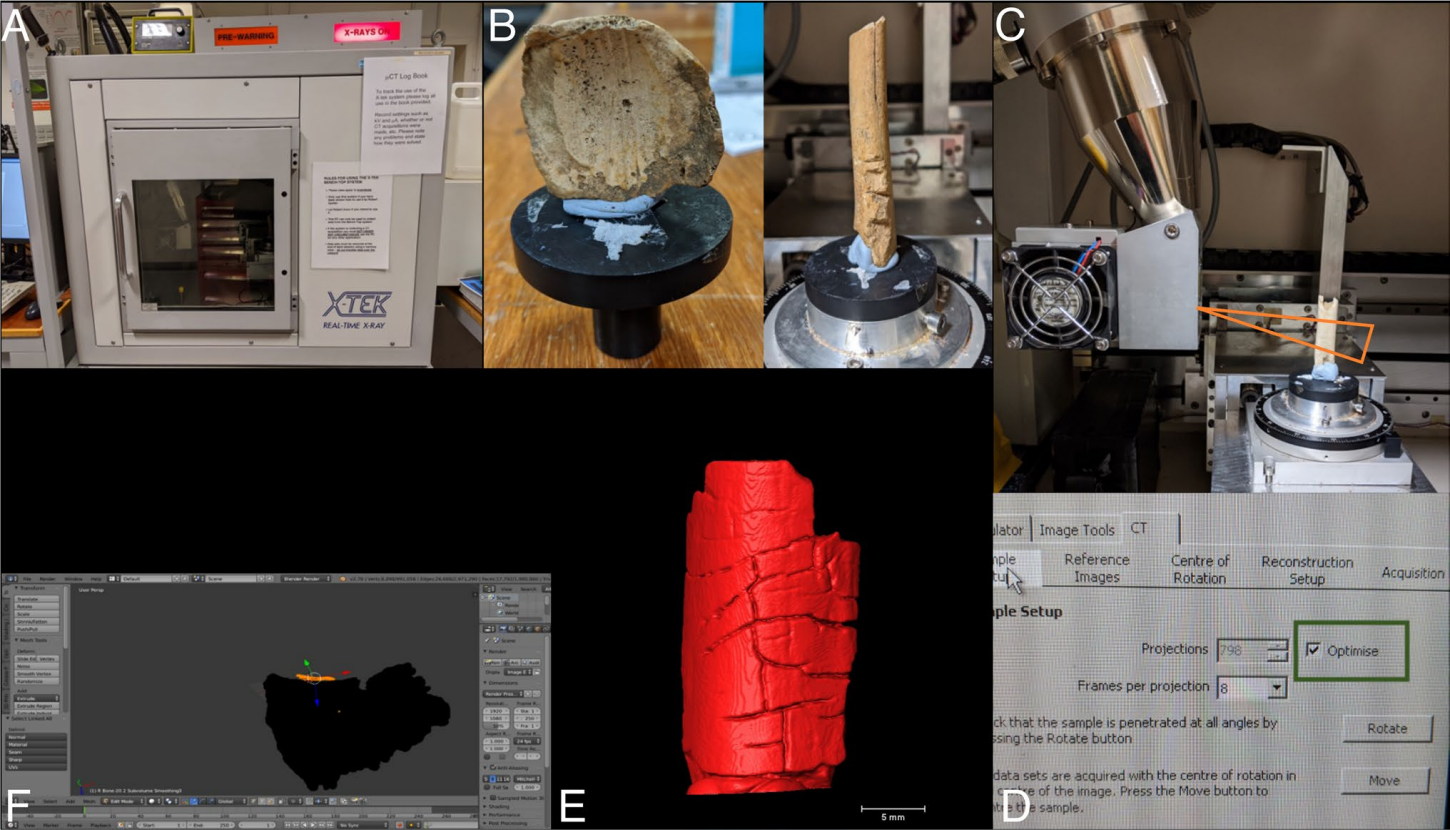
**A** X-TEK Benchtop micro-CT, main unit; **B** bones mounted on platform using Blu Tack, with platform removed from micro-CT scanner for positioning (left; sample G), and with platform in position in micro-CT scanner (right; sample A); **C** sample C in position on rotating platform in the micro-CT scanner (x-ray tube upper left), with illustration of the field of view of the bone captured by the radiation as the orange triangular ‘beam’; **D** micro-CT workstation, with ‘Optimise’ parameter selected under Sample Setup; **E** STL model of sample H viewed in Avizo; **F** deleting floating part from sample F using Blender

Each of the bones was imaged separately using an X-TEK Benchtop micro-CT scanner (Nikon Metrology, https://www.nikonmetrology.com/en-gb/). Blu Tack was used to secure the bones to the scanner platform during image capture, and in cases when a bone was too tall to fit within the field of view, a select portion of the bone was scanned (Fig. 1).

### Scan parameter validation

There are four basic parameters to consider in micro-CT imaging: X-ray tube potential (kV), X-ray tube current (µ), number of projections and number of images per projection (number of frames). kV was chosen to provide good penetration through the sample but low enough to provide a good range of transmission values. This was achieved by observing the histogram of grey values in a given projection. The combination of tube current and number of frames controls the noise in any projection. The tube current was chosen to ensure that no part of the image was saturated and the number of frames formed part of the optimisation process. To validate the number of projections selection, bone A was initially scanned with the ‘Optimise’ parameter selected in the Sample Setup menu. This selection optimises the number of projections to be acquired based upon the magnification (position of sample in the scanning geometry) chosen for the sample. Bone A was subsequently rescanned with the number of projections or number of frames adjusted at increments around the ‘Optimise’ values to identify whether the scan parameter selection altered the accuracy of the scanned model (Table 2). Following the validation results, the remaining bones were scanned using the ‘Optimise’ setting (Table 3).

**Table 2:** Micro-CT scan parameters for validation test for bone sample A (# = number of)

Model code	# projections	# frames	kV	uA
Optimised	798	8	65	45
O1	400	8	65	45
O2	600	8	65	45
O3	1000	8	65	45
O4	1200	8	65	45
O5	798	16	65	45
O6	798	4	65	45

**Table 3:** Optimised micro-CT scan parameters recorded for samples B to I (- = missing data)

Sample	# projections	# frames	kV	uA	Scan time (min)
B	896	8	65	45	13
C	868	8	65	50	14
D	1206	8	65	50	18
E	1107	8	65	50	17
F	1206	8	65	50	19
G	1206	8	65	50	19
H	720	64	60	60	-
I	720	8	65	60	-

### 3D modelling

The scan files were initially reconstructed using the built in X-TEK reconstruction software (VGI Studio software) and exported as the propriety filetype.vgi. Scan artefacts were then identified in several of the reconstructions, which were subsequently reconstructed using CT Pro 3D software to obtain optimum reconstructions.

Following reconstruction, 3D models were generated from each scan file. 3D modelling was performed in Avizo version 2019.4 (Thermo Fisher Scientific, Waltham, MA, USA), using the surface model generation tool with semi-automatic segmentation using thresholding, and a smoothing value of 3. The thresholding level was determined by one user who balanced inclusion of sufficient detail with minimal background noise (as reported by Collings and Brown [16]). Part of the bone model was eliminated during segmentation to remove the Blu Tack from the 3D model. Each of the generated surface models was exported as (ascii) stereolithic (STL) files that were suitable for 3D printing.

### Mesh comparison

The 3D models made from Bone A during the validation test (models O1 to O6) were compared to the optimised model through a mesh comparison to quantify any potential changes from the adjusted scan parameter selections. Open-source mesh comparison software CloudCompare [27], was used with the ‘Alignment and distance calculation method’ [28]. Each bone A model (O1 to O6) was initially manually aligned to the optimised model (the reference model) using the ‘Translate/Rotate’ tool, followed by automatic alignment using the fine registration tool (employing the Iterative Closest Point (ICP) algorithm) with the theoretical overlap set to 100%. After alignment, the ‘cloud-to-mesh distance’ tool was utilised to obtain the mean distance values.

### 3D printing

The STL files for each bone (A to I) were 3D printed using Selective Laser Sintering (SLS) as recommended by Carew et al. [4]. This print method is advantageous as builds are supported within the print chamber by unfused powder material and therefore additional support scaffolding is not required, thus allowing full visibility of the ‘true’ features. Prints were created using an EOSINT P100 (EOS GmbH Electro Optical Systems, Germany) printer with a white powder material (PA2200, nylon 12), at a layer height of 0.1 mm layers.

### Digitising the 3D prints

To facilitate a mesh comparison between the original bone models and the subsequent 3D prints, the 3D printed replicas were digitised using micro-CT. Thus, the 3D printed replicas of bones A–I were imaged using the same micro-CT scanner as previously, with the ‘Optimise’ scan parameter selected (Table 4). Double-sided sticky tape was used to secure the prints to the scanner mount, due to the difficulty found in removing the Blu Tack from the previous scans. The scan data from the 3D prints were reconstructed and 3D modelled as described in the “3D modelling” section, while, in this case the 3D print scan files were modelled using a smoothing factor of 5, which is the default value, which was due to independent user selection. Ideally, the smoothing factors used would have been the same; however, this was completed independently due to restrictions around Covid-19.

**Table 4:** Micro-CT scan parameters for 3D prints A–I using optimised parameters

Sample	# projections	# frames	kV	uA
A	867	8	65	50
B	830	8	65	50
C	540	8	65	50
D	665	8	65	50
E	776	8	65	50
F	1206	8	65	50
G	1206	8	65	50
H	774	8	65	50
I	558	8	65	50

### 3D print mesh comparison

The STL models from the 3D print digitisation were compared with the comparative original bone scan models to determine the accuracy of the 3D printed replicas using a mesh comparison. The mesh comparison was as described in the “Mesh comparison” section using CloudCompare, with the original bone models being the ‘reference models’ in a manner akin to Robles et al. [29].

### Quality control step

The 3D printed replica bones were checked to confirm that no errors, such as a scaling error or missing slices, had occurred, as recommended by George et al. [23]. A set of linear length and width measurements were taken from each the original bone 3D models through loading into 3D Slicer (Version 4.8.0, 3D Slicer, Brigham Women’s Hospital, Boston, MA, USA) [30] using the Ruler tool, and directly from each of the 3D printed bones using digital sliding callipers, both precise to 0.01 mm.

### A qualitative assessment

The 3D prints were visually compared with the original bones, as performed by Carew et al. [11]. In particular, the morphological details relevant to pathology, trauma and taphonomy were inspected for their congruence.

### Objective visual comparison

A quantitative visual comparison was performed between the original bones A–I and the 3D printed versions using the customised ranked scoring method from Edwards and Rogers [19], (Table 5). For a 3D print to be scored as an accurate replica, a total score of 16 or more is needed, or an individual category score of four or more [19].

**Table 5:** Print surface quality scoring method from Edwards and Rogers [19]

Scores	Description
General morphology (basic shape of subject)	
1	No similarity in basic morphological shape
2	Little to no visible resemblance in one or more areas
3	Some visible resemblance in one or more areas
4	Some visible resemblance in all areas
5	Clear and definite resemblance in overall shape
Detailed morphology (landmarks, individuated features)	
1	No similarity in detailed morphological features
2	Little to no visible resemblance in detailed morphological features
3	Some visible resemblance in one or more areas
4	Some visible resemblance in all areas
5	Clear and definite resemblance in detailed morphology
Texture (porosity, rugosity, or smoothness)	
1	No textural resemblance
2	General similarity in rugosity
3	General similarity in porosity
4	General similarity in both porosity and amount of rugosity or smoothness
5	Clear and definite resemblance in all textural similarities
Fracture pattern (fracture line appearance — visibility of lines, extension, depth)	
1	No similarity in fracture pattern/fracture pattern not visible
2	Similarity in general pattern shape only
3	Similarity in fracture line visibility and depth
4	Generally similar in overall fracture pattern
5	Clear and definite resemblance of overall fracture pattern

The scoring category of ‘Fracture Pattern’ was not always applicable to every bone, therefore different surface features were evaluated when fracture lines were not available (detailed in Table 6). Analysis was performed using both the full method with adjusted features, and using the original method but with the ‘Fracture Pattern’ element removed, as previously performed by Carew et al. [11]. This latter method therefore used accuracy cut-off values of 12 instead of 16 for the total score. If the total score per sample was the same as or greater than the cut-off value, then the sample is considered as accurate. As a result of bone I sustaining severe damage after being scanned, photographs of bone I were used to support the surface comparison together with the fragments of bone I.

**Table 6:** Features assessed on samples A–I for the fracture pattern element of the surface scoring assessment

Sample	Feature
A	Fracture line on surface
B	Tooth marks
C	Fracture line on surface
D	Fracture line on surface
E	Healing fracture line
F	Root etching on ectocranial surface
G	Eburnation grooves
H	Cremation transverse fractures
I	Fracture line through the surfaces

### Data analysis

Distance values from the mesh alignment process in CloudCompare were reported as root mean square (RMS) distances, and the mesh comparison provided mean distance values with a standard deviation [31]. Colour scalar maps and a histogram depicting the mesh distances were also recorded for each comparison. The metric differences between the digital data and the manual data were calculated (original model data minus 3D print data), and a paired t test was employed to seek any statistically significant differences following the Shapiro–Wilk test for normality (as in Carew et al. [11]).

The mesh comparison distance values obtained were also converted into a percentage difference to allow for assessments that were independent of the size of the bones, as has been previously recommended [3, 12, 20]. The mesh distance values were divided by the measured value of the bone, all multiplied by 100 to become a percentage. This was conducted using both the length and width values recorded in the quality control step to obtain two sets of percentage differences. Data calculations were carried out in Microsoft Excel version 16.44 for Mac (Microsoft, Redmond, WA, USA) and statistical analyses were performed using IBM SPSS Statistics for windows (IBM Corp, Armonk, NY, USA).

## Results

**Fig. 2:**
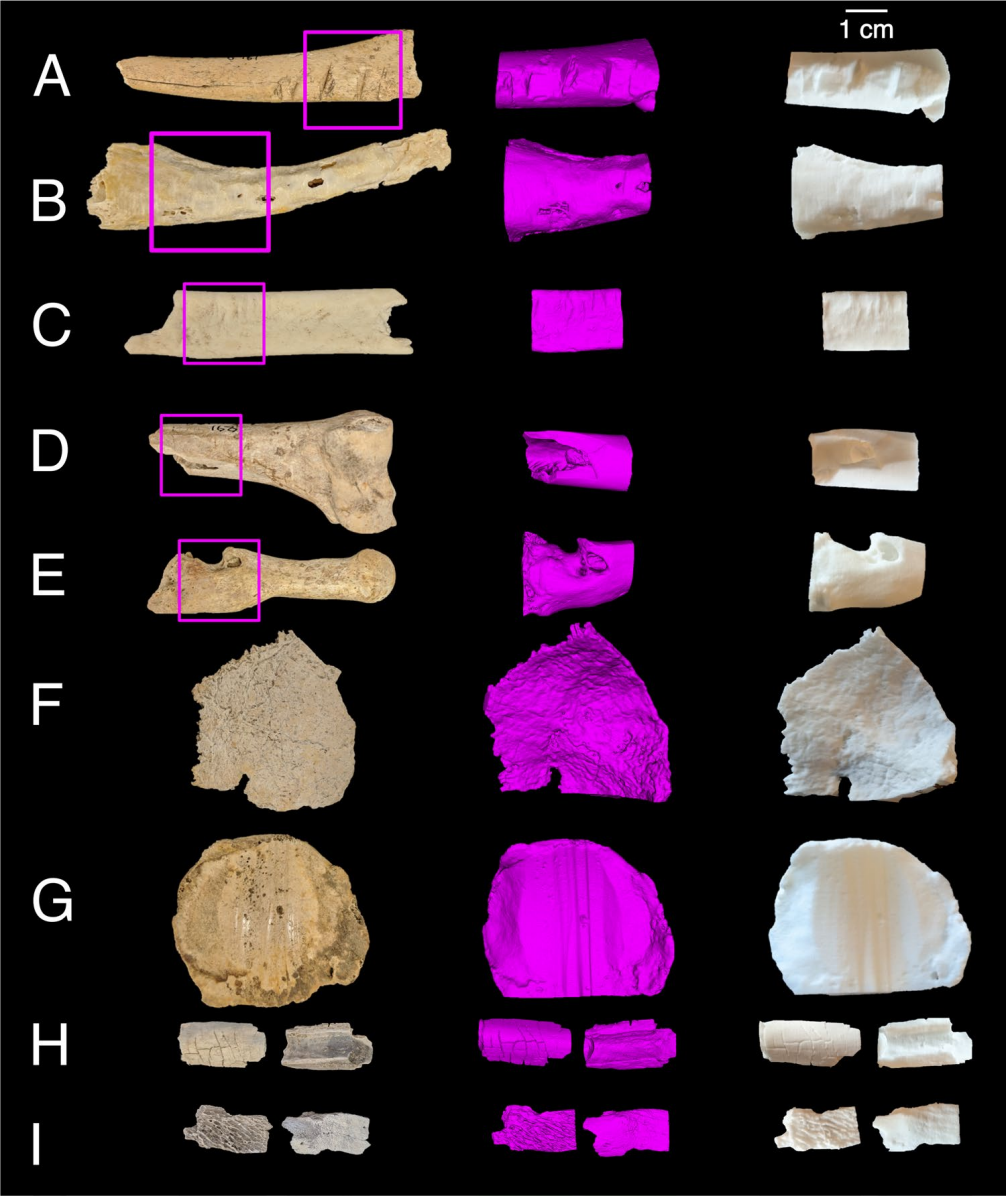
Photographs of the archaeological bones (left column) including section imaged (pink boxes), with screenshots of the corresponding virtual 3D models viewed in 3D Slicer (central column), and photographs of the SLS 3D prints (right column). Two views provided each for samples H and I. Note, 3D models and prints approximate to scale provided

Each of the nine bones was imaged using micro-CT, a 3D model generated from the scan data, and then an SLS 3D print generated from each model (Fig. 2).

### Scan parameter validation

**Fig. 3:**
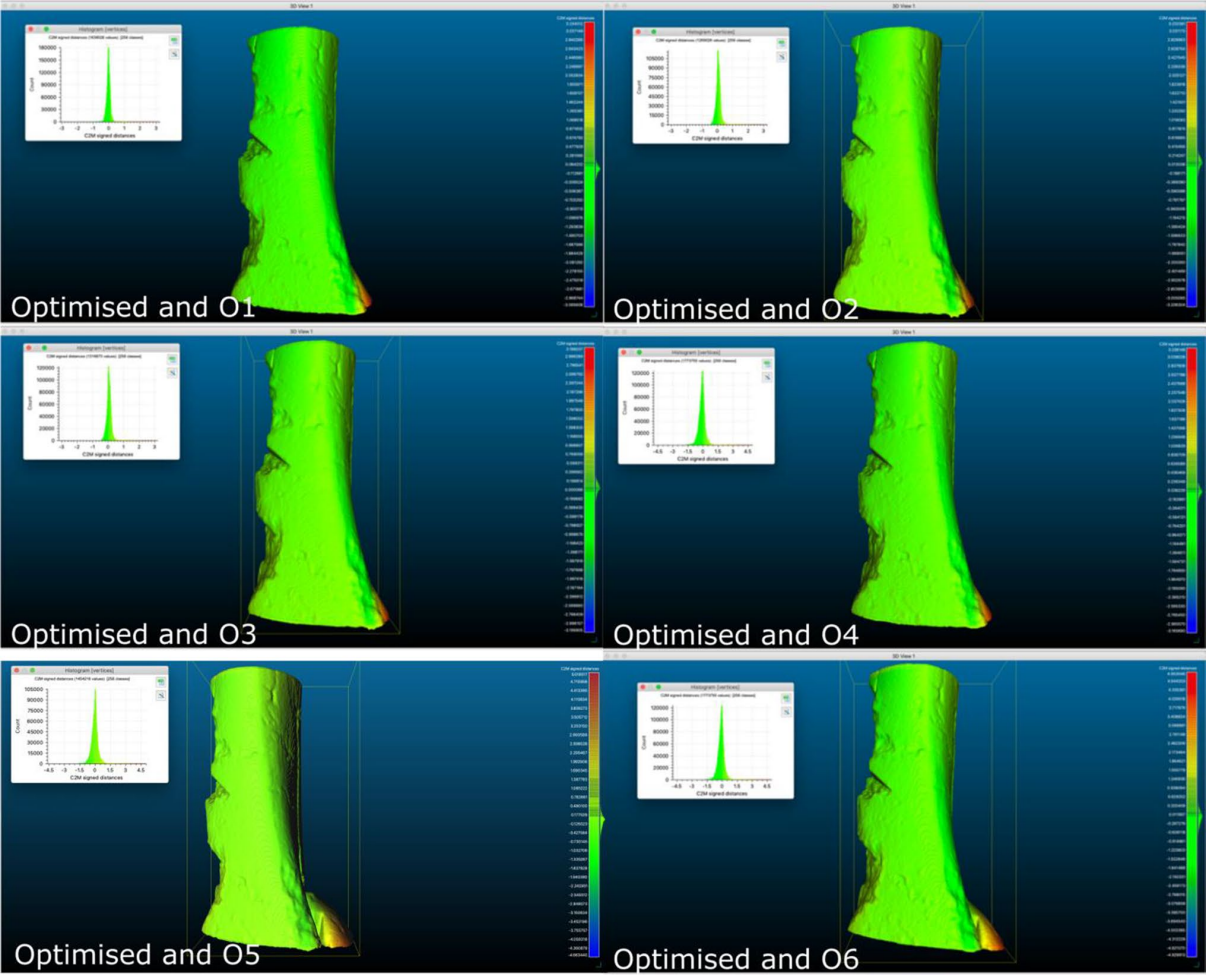
Colour map showing mesh comparison distances when compared with the ‘Optimised’ setting model, for models O1–O6 (sample A)

The optimised 3D model of bone A was compared against models O1-O6 (that had parameters adjusted around the optimised setting). The alignment of the models resulted in satisfactory alignments (Table 7) with RMS values less than 0.1 mm in each case (ranging from − 0.06 to 0.09 mm). Very little distance can be observed on the colour maps or histogram plots comparing the models (Fig. 3), except in the areas that contained the Blu Tack.

**Table 7:** CloudCompare distance values (mm) (SD, standard deviation)

Model	RMS	Mean distance	SD
O1	0.26	0.00	0.25
O2	0.29	0.09	0.28
O3	0.29	0.08	0.28
O4	0.26	0.03	0.26
O5	0.54	0.03	0.55
O6	0.52	− 0.06	0.52

### Quality control step

Linear measurement data taken from the original 3D bone models were compared against data recorded from the 3D printed replicas, and the difference between the two calculated (Table 8). Length and width measurements were all less than 1.0 mm. Shapiro–Wilk tests for normalcy indicated that the datasets were all normally distributed (*p*-values > 0.05) and paired t-tests indicated that there were no statistically significant differences between the datasets (*p*-value 0.6 for length data and 0.22 for width data).

**Table 8:** Length and width measurement data recorded from the original 3D (STL) models and the 3D printed bones, with the difference between the two calculated (original model data minus 3D print data) (mm)

Sample	Length			Width		
	Original 3D Model	3D Print	Difference	Original 3D Model	3D Print	Difference
A	36.79	37.50	− 0.71	12.59	13.30	− 0.71
B	34.96	34.70	0.26	10.01	10.00	0.01
C	19.81	20.00	− 0.19	3.32	3.30	0.02
D	26.55	27.10	− 0.55	13.55	13.10	0.45
E	25.15	25.20	− 0.05	8.58	9.10	− 0.52
F	51.45	51.00	0.45	4.01	3.60	0.41
G	34.00	34.80	− 0.80	15.68	15.90	− 0.22
H	26.77	26.30	0.47	3.70	4.50	− 0.80
I	23.91	23.60	0.31	3.95	4.40	− 0.46

### Qualitative assessment

**Fig. 4:**
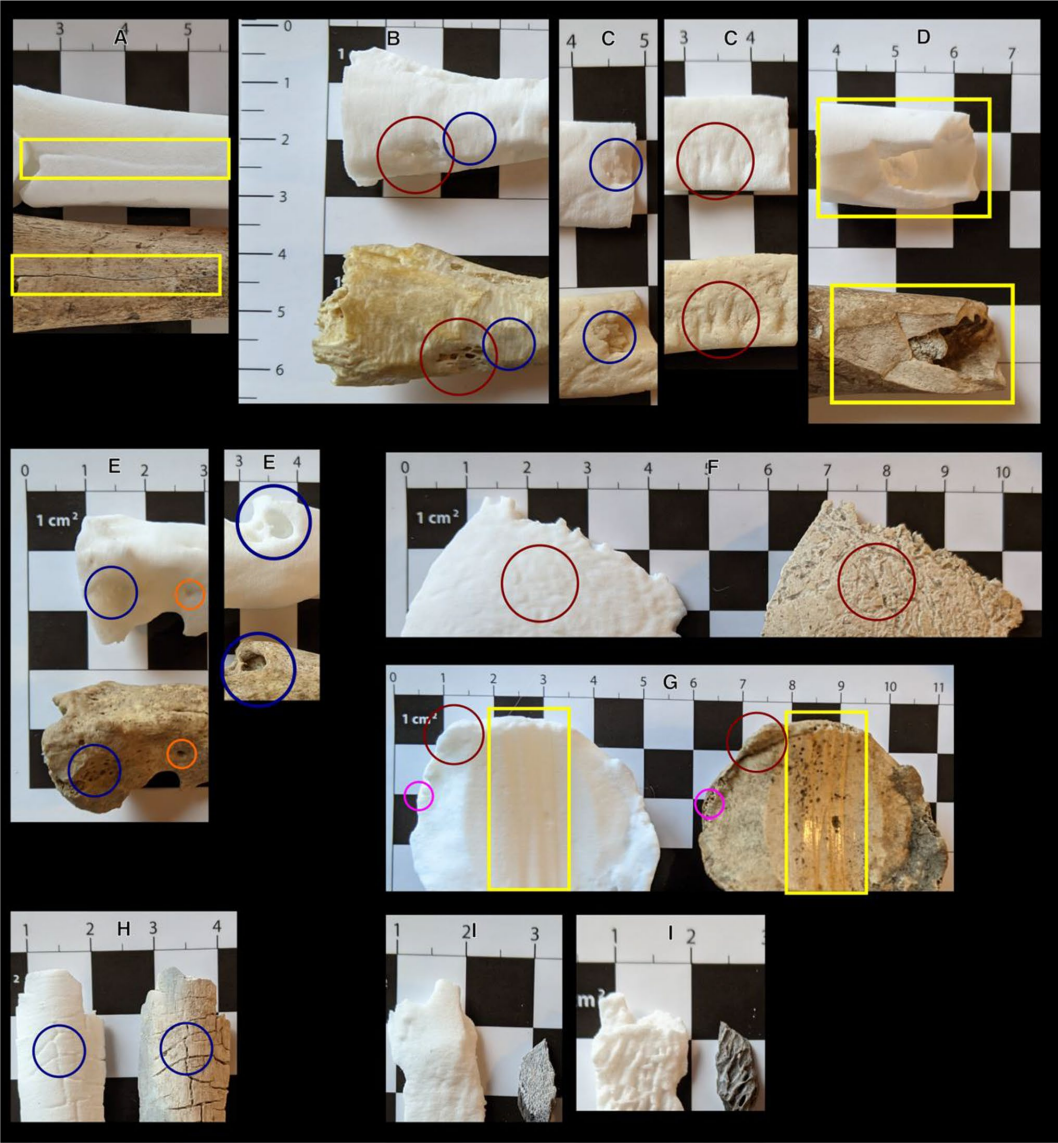
Comparisons of 3D prints to original bone samples: Sample A, fracture line visible in yellow rectangular box; Sample B, exposed trabecular bone in larger red circle, toothmarks in smaller blue circle; Sample C, (left image) depressed fracture in smaller blue circle and (right image) toothmarks in larger red circle; Sample D, butchery/chop mark in yellow rectangular box; Sample E, (left image) macroporosity in larger blue circle and smaller orange circle, and (right image) pathological remodelling features in larger blue circle; Sample F, endocranial root etchings in large red circle; Sample G, exposed trabecular bone in smaller pink circle, osteophytes in larger red circle, and eburnation and grooves in yellow rectangular box; Sample H, transverse fractures in blue circle; Sample I, detail on external surface (left image) and features on internal surface (right image). Scales in cm

An assessment of congruence between the original bones and the 3D printed replica bones was performed, detailed in Table 9. Overall, this qualitative assessment indicated that the contrast between natural colour, texture, and depth of the surface micromorphological details was often lost with the monochrome white colour of the prints (see Fig. 4 for examples).

**Table 9:** Qualitative observations from a visual comparison between the 3D printed bones and the original bones (samples A–I)

Sample	Qualitative observations
A	Butchery cut marks (c. 0.1–2.7 mm) visible but fine details lost on print. Fracture line visible, but as a continuous groove rather than a discontinuous separation. Periostitis/woven bone detail lost on print
B	Exposed trabecular bone well-presented. Rat toothmarks were somewhat presented, with loss of detail and depth of grooves
C	Flaking/depression fracture fairly well presented with some loss of detail; root etching visible, toothmarks well presented
D	Butchery/chop mark and exposed trabecular bone were well represented with defined edges; very shallow root etching lost on print
E	All features well-presented including macroporosity, microporosity generally lost
F	Endocranial root etchings generally well-presented, finer ectocranial root etchings losing detail. Exposed diploë (spongy) bone not observable, sutures visible, fractured edges fairly well-defined
G	Osteophytes, enthesophytes, exostosis, and woven bone all well-presented. Eburnation and grooves on anterior surface also well-presented, but with loss of smoothness and shine. Exposed trabecular bone visible but definition lost
H	Transverse fractures were well presented, with similar depth and appearance. Little-to-no loss of detail
I	Features on internal surface were well-presented; fine details on the external surface were lost

### Objective visual comparison

**Fig. 5:**
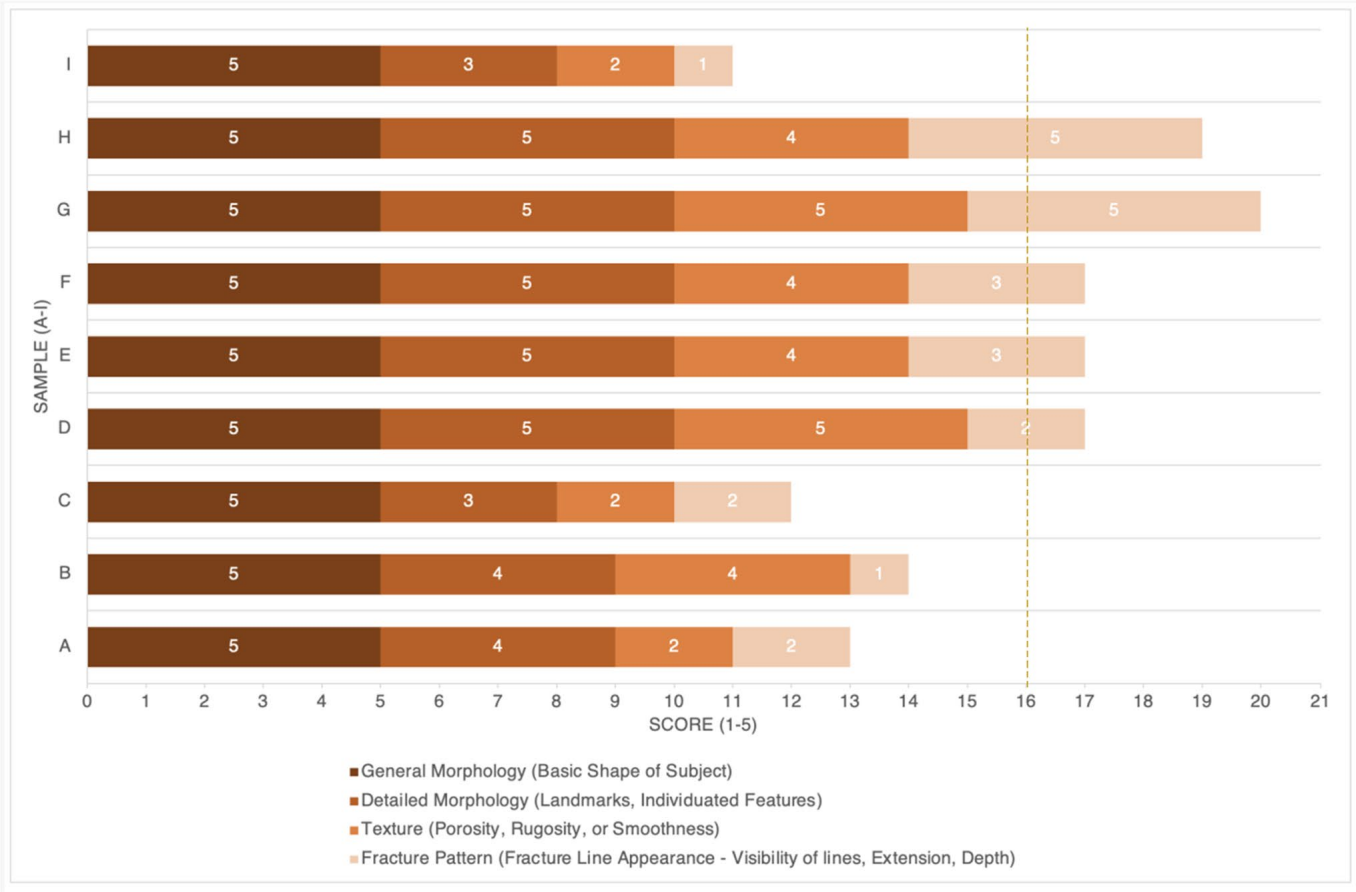
Stacked bar chart illustrating the quantitative scores obtained with samples A–I (dashed line representing the cut-off total score value for determining the prints as accurate). The adjusted scoring method without the fracture pattern element resulted in accurate scores for six of nine 3D prints (B and D to H), where each print scored greater than the adjusted cut-off value of 12

The full customised surface scoring method was applied to compare the 3D printed bones to the original bones. The quantitative surface scores from five of the nine samples (bones D to H) indicated that these five prints were accurate (Table 10). The fracture pattern scoring category resulted in lower scores than the other categories (Fig. 5), and the general morphology category consistently scored all prints as the highest score of 5.

**Table 10:** Quantitative surface scores using Edwards and Rogers [19] with samples A–I. * = prints or features scored as accurate

Score/criteria	A	B	C	D	E	F	G	H	I
General Morphology	5*	5*	5*	5*	5*	5*	5*	5*	5*
Detailed Morphology	4*	4*	3	5*	5*	5*	5*	5*	3
Texture	2	4*	2	5*	4*	4*	5*	4*	2
Fracture Pattern	2	1	2	2	3	3	5*	5*	1
TOTAL SCORE	13	14	12	17*	17*	17*	20*	19*	11

### 3D print mesh comparison

**Fig. 6:**
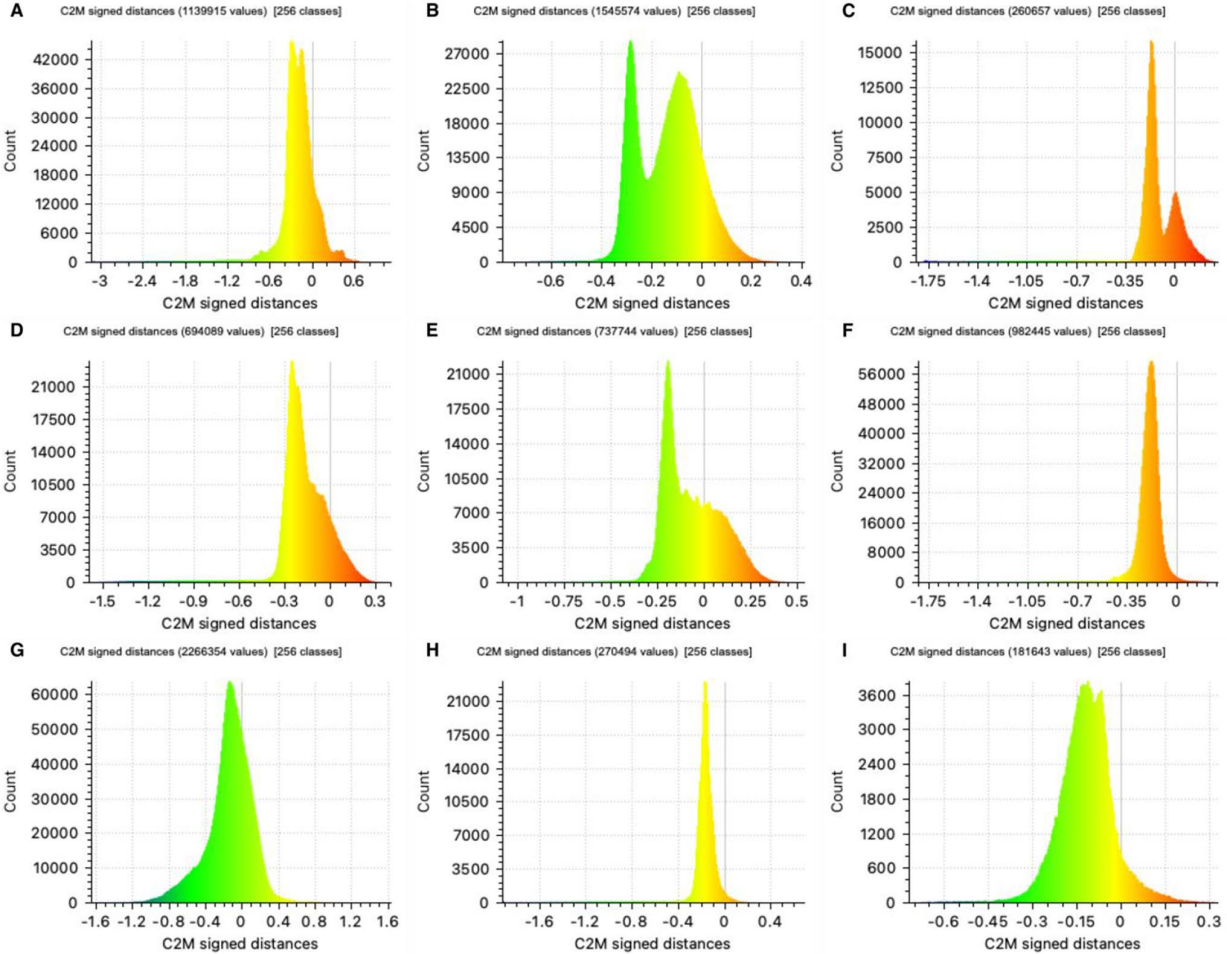
Histograms for each mesh comparison for samples A through I, illustrating the distribution of the mesh distance values (C2M = cloud to mesh). Scales are model specific in each histogram

The digitised 3D printed bone models were compared to the original bone models through a mesh comparison. The values from the alignment process and the distance computation are provided in Table 11. The average RMS value from the alignment process was 0.24 mm, which ranged from 0.15 to 0.36 mm. Mean distance values ranged from − 0.2 to − 0.07 mm, which were all within the ideal accuracy threshold of ± 1.0 mm [11]. Histograms of the mesh distance values (Fig. 6) illustrated that the spread of the deviations peaked at or around zero and tended to give more negative values than positive.

**Table 11:** Mesh comparison results from original 3D scan model versus 3D print scan model in CloudCompare; RMS (root mean square), mean distance and std deviation (standard deviation) (mm)

Sample	RMS	Mean distance	Std deviation
A	0.36	− 0.20	0.27
B	0.18	− 0.13	0.12
C	0.27	− 0.14	0.20
D	0.26	− 0.17	0.17
E	0.17	− 0.07	0.15
F	0.23	− 0.20	0.10
G	0.28	− 0.13	0.24
H	0.26	− 0.18	0.16
I	0.15	− 0.12	0.09
Average	0.24	− 0.15	0.17

The mean distance values were converted into percentage differences to give a non-dimensionalised value. Employing the length data resulted in percentage differences of − 0.3 to − 0.7% (± 0.2 to 1.0) and using the width values resulted in mean percentage differences from − 0.8 to − 4.9% (± 1.2 to 6.0).

## Discussion

Verification of the accuracy of 3D printed replicas is an important step in crime reconstructions. Moreover, the representation of intricate details from process such as trauma, taphonomy and pathology needs to be reliable for evaluative interpretation and courtroom demonstration purposes. This multi-method experimental approach study utilised nine bone samples exhibiting various types of micromorphological details. A preliminary scan parameter validation test was conducted to validate the use of the Optimise setting for the micro-CT scans which showed very low distance values between the models scanned at varying parameters through a mesh comparison. Adjusting the scan parameters did not have an observable effect on the size of the 3D models, which therefore justified the use of the ‘Optimise’ setting for the subsequent bone imaging.

A quality control step (step 1) examined the overall size of the 3D printed bones. The 3D prints were identified to be within ± 1.0 mm of the original bone 3D models, with no statistically significant result found between the manual and virtual measurement data. These results established the overall accuracy of the 3D printed bones and confirmed that no scaling error had occurred. These results were congruent with those obtained by Carew et al. [11] who used a similar quality control technique, although using a clinical CT scanner not micro CT. The qualitative assessment (step 2) established that fracture lines were observable on the 3D printed surfaces, but that these lines often presented as continuous grooves, rather than discontinuous separate margins. Exposed trabecular bone and toothmarks were also generally well-presented on the prints, but with loss of detail or depth often identified. Generally, the visual comparison found that the 3D printed bone replicas adequately represented the micromorphological details, whereby the features were usually observable. However, there was often loss of detail, depth, and fine texture, such as the shine from eburnation found on bone G (the patella). Factors such as the scan parameters, the level of smoothing applied during modelling and the resolution of the printer will all influence the presentation of the micromorphological details. As discussed, each of these needs careful consideration and further research to explore and quantify these effects.

Additionally, it was noted that the contrast between the colour, texture and depth was sometimes missing on the 3D prints, primarily due to the replication using a monochrome white material. Shading and colour cues are necessary for monocular vision processing [32], but this factor cannot be replicated with monochrome prints. It may be possible to produce multicoloured 3D prints using photorealistic printing from photographs coupled with surface or volumetric scanning [31], however, this would only be an accurate colour representation if captured from dry or exposed human remains. Further, this photorealistic aspect could mean that the 3D printed replicas become too realistic or too disturbing, as with colour photographs [33, 34]. It is therefore proposed that perhaps the loss of detail found with a monochrome 3D printed replica is an accepted loss that facilitates the use of the 3D print as a less-graphic, sanitised option for courtroom demonstrations.

An objective visual comparison (step 3) utilised the surface scoring method developed by Edwards and Rogers [19] to quantitatively score the surface quality of the printed bones. All of the 3D printed bones obtained the highest score of 5 for the general morphology category, which corresponds to the quality control step, that each of the prints had accurate overall morphology. High scores were also assigned to the detailed morphology category, but with lower scores assigned to the texture and fracture pattern categories. These findings echo those presented by Carew et al. [11], who also found 3D prints to be accurate overall, but limited by loss of finer details. This visual comparison identified that five of the nine bones (D to H) exhibited accurate surface representations overall. Bone B was scored as accurate when the altered method excluding fracture pattern assessment was used. Previous research by Carew et al. [11] utilising clinical CT scanning, identified only two of their nine prints to be accurate from this scoring method. Therefore, this study presents potential improvement in accuracy rates, with five of the nine prints scored as accurate using the micro-CT scanning technique; this is despite the bones used in this study having more complex micromorphology than the previous study. The scoring method worked well as an adaptable objective recording method. However, the terminology ‘resemblance’ in the detailed morphology category is somewhat ambiguous, and left room for dissimilarity.

Finally, the quantitative assessment (step 4) used a mesh comparison to assess the accuracy of the digitised 3D print models. The initial alignment process produced low RMS values, indicating close alignments between the tested models. The scanned 3D printed bones were then found to be accurate to the original bone models to within the ideal threshold of ± 1.0 mm. Moreover, the prints were accurate to within 0.2 mm using the mean distance values indicating a high degree of accuracy. This result is similar to other mesh comparison results reported between 3D bone models, such as Smith et al. [35] (0.1 ± 0.1), Baier et al. [3] (less than 0.62 mm), and Robles et al. [29] (± 0.4 mm). Conversion of the mesh distance values to percentages provided non-dimensionalised values to indicate the accuracy of the prints. The percentage mean differences were approximately 0–1% for length and 0–5% for width. These values again indicated low differences and good congruency between the original bone models and the digitised 3D prints, similar to those previously reported [3, 12]. These values are independent of the size of the sample and provide a valuable figure for forensic reporting purposes that may be more meaningful for lay interpretation.

The majority of the mesh distance values reported were also identified as coming from the internal bone structure, not from the bone surface. Furthermore, while the mesh comparison method is a simple, effective method for comparing model dimensions, the method does not effectively identify differences in surface quality. As seen in this study whereby high model accuracy was obtained, but differences were also seen in the presentation of micromorphological surface details. These findings concur with those from Edwards and Rogers [19], who note that distance values do not necessarily reflect the quality of printed replica, as any morphological differences will be minimal irrespective of digitisation method used. The implementation of visual inspection methods is vital for proper examination of print quality, and mesh comparison methods alone should not be used.

### Implications for practice

The findings from this multi-method study have supported the use of 3D printed bones from micro-CT reconstructions for forensic purposes. The level of detail obtained on the 3D printed replicas was sufficient for overall morphology and most micromorphological features, but with loss of detail around the contrast between the colour, texture, and depth. However, the details that are not-observable may not be important for evaluative interpretation by jurors in a courtroom. In this case, the print may be supported by concurrent presentation with a digital 3D render as previously recommended [4, 11]. This is coupled with the premise that a 3D printed replica ought to be demonstrably accurate to a given degree through quality control and accuracy checks. A series of steps to test and confirm the accuracy of 3D printed bones is outlined:

- 1. Print accuracy verification (using metric methods)
- 2. Qualitative inspection (print to original comparison)
- 3. Quantitative surface scoring (print to original comparison)
- 4. Quantitative mesh comparison (if additional measure of accuracy needed)
- 5. Scan parameter validation (if needed, specific for micro-CT)

These steps are a user-friendly approach that can be adapted for use and performed using open-source software providing an accessible approach suitable for use in academic or practice settings. The approach is also adaptable depending on the imaging technique used and does not constrict users to a finite accuracy figure. The determination of a 3D printed replica as accurate to a given degree based upon the methods and equipment used is the important factor.

**Fig. 7:**
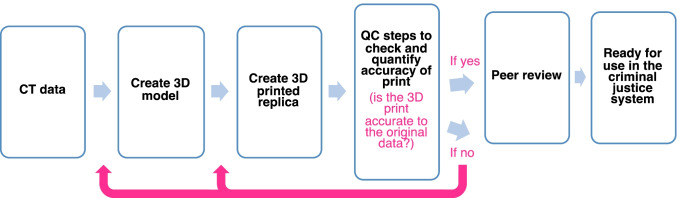
Example of a standard operating procedure incorporating the presented quality control (QC) steps. A 3D print will be re-modelled or re-printed if deemed to be inaccurate depending upon the issue

The proposed steps can be considered as part of a quality control (QC) scheme in the context of a simple laboratory standard operating procedure, to illustrate its utility within the wider development and verification process (Fig. 7). This procedure includes the decision-making of the user, for example, if a 3D printed is found to be accurate following the steps, then the print may be confirmed using peer-review and subsequently prepared for use in the criminal justice system (i.e., for courtroom demonstration). Conversely, if a 3D is not found to be sufficiently accurate following the QC steps, then the 3D model or 3D print may be reproduced (depending on the issues identified).

The assessment of the micromorphology of 3D printed bones presents an innovative approach to a novel area in forensic science. Little work has previously investigated the accuracy of fine 3D printed bone detail and the results of this study build upon new data and add valuable data to the literature. Further, the multi-method approach and the steps outlined in Fig. 7 present new tools that forensic actors can use in practice.

## Conclusion

The multi-method experimental approach employed in this study has generated insights that contribute towards the articulation of a pathway for assessing the accuracy of the surface of 3D printed bones. The results identified that:

- The quality control step identified the 3D printed bones to be accurate to the original bones to within 1.0 mm
- The qualitative assessment found good representation of surface features from trauma, taphonomy and pathology, but intricate details, depths, and fine textures were sometimes lost
- Five of the nine 3D printed bones were scored as accurate using the objective visual comparison method
- The quantitative assessment resulted in mesh comparison distances to within 0.2 mm (0–1% based on length and 0–5% based on width) between the original 3D models and the digitised 3D prints

The pathway to check for print quality can be used in future crime reconstruction approaches to protect the integrity of the final 3D printed replicas for forensic applications. It should be considered in the wider forensic science framework, considering factors such as accreditation, certification, standardisation, and the guidance from the Forensic Science Regulator in the UK. This presents an opportunity to develop systems for producing robust printed reconstructions for forensic applications. These findings complement the growing body of published literature supporting the use of 3D printed bones in forensic anthropology crime reconstructions [3–5, 11, 16, 20] and can be incorporated into emerging forensic 3D printing guidance.

## Data Availability

All data generated during this study are included in this published article.
